# Toxicologic Assessment of a Commercial Decolorized Whole Leaf Aloe Vera Juice, Lily of the Desert Filtered Whole Leaf Juice with Aloesorb

**DOI:** 10.1155/2013/802453

**Published:** 2013-03-11

**Authors:** Inder Sehgal, Wallace D. Winters, Michael Scott, Andrew David, Glenn Gillis, Thaya Stoufflet, Anand Nair, Konstantine Kousoulas

**Affiliations:** ^1^LSU School of Veterinary Medicine, Skip Bertman Drive, Baton Rouge, LA 70803, USA; ^2^Comparative Biomedical Sciences Department, LSU School of Veterinary Medicine, Skip Bertman Drive, Baton Rouge, LA 70803, USA; ^3^Science, Technology and Toxicology (ST&T) Consultants, 655 Montgomery Street, Suite 800, San Francisco, CA 94111, USA; ^4^Division of Biotechnology and Molecular Medicine (BIOMMED), LSU School of Veterinary Medicine, Skip Bertman Drive, Baton Rouge, LA 70803, USA; ^5^Lily of the Desert, 1887 Geesling Road, Denton, TX 76208, USA

## Abstract

Aloe vera, a common ingredient in cosmetics, is increasingly being consumed as a beverage supplement. Although consumer interest in aloe likely stems from its association with several health benefits, a concern has also been raised by a National Toxicology Program Report that a nondecolorized whole leaf aloe vera extract taken internally by rats was associated with intestinal mucosal hyperplasia and ultimately malignancy. We tested a decolorized whole leaf (DCWL) aloe vera, treated with activated charcoal to remove the latex portion of the plant, for genotoxicity in bacteria, acute/subacute toxicity in B6C3F1 mice, and subchronic toxicity in F344 rats. We found this DCWL aloe vera juice to be nongenotoxic in histidine reversion and DNA repair assays. Following acute administration, mice exhibited no adverse signs at 3- or 14-day evaluation periods. When fed to male and female F344 rats over 13 weeks, DCWL aloe led to no toxicity as assessed by behavior, stools, weight gain, feed consumption, organ weights, and hematologic or clinical chemistry profiles. These rats had intestinal mucosal morphologies—examined grossly and microscopically—that were similar to controls. Our studies show that oral administration of this DCWL aloe juice has a different toxicology profile than that of the untreated aloe juice at exposures up to 13 weeks.

## 1. Introduction

Aloe vera is a common ingredient in cosmetics, skin care products, and increasingly, beverages and food products [1]. Recent consumer interest in aloe beverages may stem from the association of aloe juice with a variety of both anecdotal and experimental research-supported health benefits including the prevention or treatment of various tumors [2, 3], arthritis [4], diabetes [5], enhanced immunity [6], and decreased cholesterol levels [7].

Aloe juice is approximately 99% water [8] and the remainder consists of minerals, vitamins, polysaccharides, lipids, phenolic compounds, and organic acids. According to the International Aloe Scientific Council, the aloe leaf can be processed into two types of juices for commercial use: inner leaf gel juice and decolorized whole leaf juice [9]. Inner leaf gel juice is produced from only the gelatinous fillet of the leaf. Decolorized whole leaf juice is produced by grinding the leaf followed by treatment of extracted juice with activated charcoal to remove aloe “latex” [10]. Charcoal treatment is necessary since the latex, which exists as a separate liquid between the outer rind and inner fillet gel, contains bitter phenolic molecules including anthraquinone C- and O-glycosides, anthrones, and some free anthraquinones [11]. The major C-glycoside, aloin A, is the major anthraquinone in aloe latex and when oxidized, yields aloe-emodin, a free anthraquinone [11]. Anthraquinones are associated with diarrhea *in vivo* [12, 13], weight loss, gall bladder lesions, renal tubule pigmentation, and renal tubule hyaline droplets [14]. *In vitro*, anthraquinones cause mutations in mouse lymphoma [15] and salmonella assays [16]. Aloe beverages, which follow IASC standards contain less than 10 ppm aloin A and its isomer, aloin B [9].

Toxicology of various aloe products has been examined *in vivo*, and these studies collectively suggest that the potential for toxicity depends on the plant sections used in juice production. Oral consumption of an ethanol extract of whole leaf aloe over 3 months by mice resulted in reproductive toxicity and increased mortality [17]. However, subchronic administration of aloe taken from the inner leaf gel resulted in no evidence of oral toxicity to rats [18]. In 2010, the National Toxicology Program [10] released a technical report on aloe vera that concluded that “there was clear evidence of carcinogenic activity of a nondecolorized whole leaf extract of Aloe vera in male and female F344/N rats based upon increased incidences of adenomas and carcinomas of the large intestine.” The results of this report have been referred to on various health-related websites suggesting caution in consumption of aloe products [19–22] and have generated substantial interest and concern by aloe producers, because the study results were based on a nondecolorized whole leaf extract that is not commercially available [23]. The NTP study was designed to deliver nondecolorized whole leaf, freeze-dried juice powder to test animals through their drinking water at levels up to 3.0% (wt/wt), and the study examined potential toxicologic effects of aloe in both B6C3F1 mice and F344 rats over time periods of 14 days, 13 weeks, and 2 years [10].

While an increased incidence of tumorigenesis was observed in the large intestine of rats in the 2-year study, no lesions were observed in mice or rats in acute studies; however, there was decreased water consumption in both rodent species at the highest aloe levels. During a subchronic period (13 weeks), mice administered aloe were similar to controls in most parameters with the exception of increased incidences of mucosal hyperplasia with goblet cell hyperplasia in the cecum and large intestine. This mucosal and goblet cell hyperplasia was also observed in rats at 13 weeks. These rats displayed increased mortalities and weight losses at the high aloe concentrations. Rats exposed for two years also were found to have mucosal hyperplasia in the colon, particularly in the cecum and ascending portions. Large intestine mucosal/goblet cell hyperplasias were also observed in mice exposed for two years; however, these mice did not show increased neoplasms and had similar survival and body weights as control groups.

The nondecolorized whole leaf powder used by the NTP contained the bitter latex of the aloe plant and therefore, the addition of this aloe directly to drinking water could have contributed to the study's outcomes. Palatability can influence water consumption and conceivably could have also contributed to the weight loss and mortality in the two-year study. Commercial aloe beverages that contain little or no latex anthraquinones may have yielded different outcomes in the subchronic or chronic exposure tests. The NTP also assessed the acute effects of aloe gel only and decolorized (activated charcoal treated) whole leaf aloe, and in these studies, mice and rat body weights, water and feed consumptions, organ weights, hematology, clinical chemistry, and urine chemistry were generally similar to controls. However, only the nondecolorized extract was used in the 13-week and 2-year studies.

Although the reported incidence of large intestinal tumors has garnered the most attention from the NTP report, the incidences of intestinal mucosal hyperplasia were the most consistent observations across species exposed both subchronically and chronically. This mucosal hyperplasia suggests a toxicological response to one or more whole leaf aloe components. In the rat, hyperplasia of both the mucosa and goblet cells may indicate cellular damage and could be prognostic of later tumor development since control rats showed little or no hyperplasia at 13 weeks or adenoma/carcinomas of the large intestine at 2 years.

To date, no evaluation of the de-colorized whole leaf Aloe vera juice has been reported. In the present study, we report the first toxicological evaluation of a commercially available, decolorized, whole leaf (DCWL) Aloe vera beverage, Lily of the Desert Filtered Whole Leaf Aloe vera Juice. This particular product can be considered as being well characterized with regard to high-molecular-weight polysaccharide content [24] and immunomodulating activity [25, 26]. Testing included assessment of potential genotoxicity *in vitro* and acute toxicity *in vivo* using a B6C3F1 mouse model. Further testing included a 13-week study utilizing F344 rats in which the DCWL juice was administered through the rat's chow in order to impart a minimal effect on palatability. Examinations included large intestinal histology to understand if aloe beverages derived from DCWL aloe vera juice produced toxicologic signs associated with nondecolorized whole leaf juice.

## 2. Materials and Methods

### 2.1. Procurement of DCWL Aloe Vera Juice

Aloe vera leaves were harvested and maintained under cold storage (8°C) until processing. Time lapse from harvest to processing was less than six hours. The harvested leaves were washed, disinfected, and macerated to yield an intermediate raw material representative of a nondecolorized whole leaf extract as was used in the aforementioned NTP study. The nondecolorized extract was then filtered using carbon/diatomaceous earth to yield a DCWL extract. To this extract, a proprietary isolate of high-molecular-weight aloe vera polysaccharide, Aloesorb, was added at a rate of 30 mg solid/fluid ounce. The resulting extract was flash-pasteurized to yield the commercial product (Lily of the Desert Filtered Whole Leaf Aloe vera Juice with Aloesorb). This product was vacuum-dried to yield a 7-fold concentrate.

NMR analysis of the resulting extract established (1) the presence of Aloe vera (by confirming the presence of aloe vera polysaccharide, malic acid, and glucose), (2) the presence of whole leaf markers (isocitrate and isocitrate-lactone), and (3) the absence of adulterants/stabilizers in the extract. HPLC analysis confirmed the presence of low levels of anthraquinones (Aloin A at 0.868 ppm, Aloin B at 1.335 ppm, and Aloe-emodin at 0.200 ppm). These results qualify the extract as a decolorized whole leaf aloe vera extract.

The juice was maintained under constant refrigeration until use. Prior to testing, the aloe juice was lyophilized and determined to be 10.65 ± 0.11% nonaqueous. It was also found to be acidic (pH = 3.9). Prior to analysis in genotoxicity assays, the aloe juice was filtered using positive pressure to render it sterile and the pH adjusted to 7.5 to accommodate *Salmonella* bacterial assays that are pH-sensitive. In order to maximize the level of aloe administered to test mice, the 7x juice was further concentrated by lyophilization to a final concentration of 35x in a Labconco lyophilizer over a 6-hour period. This concentrated juice was administered in gavage studies. A single lot (#021412-4) was used throughout the studies.

### 2.2. Genotoxicity Assays

Potential mutagenicity and/or DNA damage were assessed *in vitro* with two bacterial assays. An assay for mutagenesis was used, which is based on the Ames test utilizing *Salmonella typhimurium* strain TA100 but modified for liquid culture and a 96-well plate scale. The second assay detected potential DNA damage utilizing an *E*. *coli* strain containing a transgene for beta-galactosidase downstream of the SOS-DNA repair promoter system. Both assays were purchased in a commercial format from EBPI Bio-Detection Products (Mississauga, ON, Canada), referred to the as Muta-Chromo Plate and SOS-Chromo Test Assays, respectively.

To test for potential metabolic generation of mutagens, DCWL aloe vera juice was also tested in the presence of S9 liver extract. For testing with *Salmonella*, the juice was combined with a reaction mix containing growth substances and a pH indictor. The juice was administered at 21x, 14x, and 7x concentrations. As a positive control for direct acting mutagenesis (i.e., independent of metabolic conversion), either sodium azide (0.38 *μ*M final concentration) or 2-nitrofluorene (7.1 *μ*M final concentration) was included in one plate. 2-Aminoanthracene (2.60 *μ*M final concentration) was used as a positive control for mutagenesis requiring metabolism. Plates containing no aloe juice were used to measure spontaneous mutations over the incubation periods, and these blanks were compared to all aloe-containing plates. The number of positive mutated wells was scored on days 3, 4, and 5 of growth. These numbers were then compared statistically to blanks.

For the DNA damage assay in *E. coli*, the DCWL juice was tested at levels of 21x, 14x, 7x, 3.5x, 1.75x, 0.88x, 0.44x, 0.22x, 0.11x, and 0.055x. When diluted, the juice was suspended in sterile 10% dimethyl sulfoxide (DMSO) in sterile 0.85% saline. A positive control for DNA damage independent of metabolism was included (4-nitroquinoline oxide [4NQO]) and used at levels of 10.0 *μ*g/mL (52.6 *μ*M), 5.0, 2.5, 1.25, 0.625, and 0.3125 *μ*g/mL. A positive control for mutagenesis requiring metabolism was included in another plate (2-aminoanthracene) and used at 100.0 (2.6 *μ*M), 50.0, 25.0, 12.5, 6.25, and 3.125 *μ*g/mL. DNA damage was quantitated spectrophotometrically by color development. Data are expressed as the mean and standard deviation of the SOS induction.

### 2.3. Animals

All mice were purchased from Jackson Laboratories, Inc. and were ordered between 4 and 6 weeks of age. After a two-week period of quarantine, the mice were numbered for identification by ear tag. Upon initiation of studies, mice were weighed, sorted into boxes of 5 mice each, and then randomly assigned to control or aloe juice groups. Mice were housed within the Louisiana State University Division of Laboratory Medicine's AALAC-approved vivarium in Super Mouse Micro Isolation 750 boxes that are racked in an Enviro-Gard-B microisolation control cage rack (Lab Products, Seaford, DE). Cage changes with fresh litter and fresh water were provided weekly to the mice. Mouse boxes were stocked with clean/autoclaved Bio-Serv (Frenchtown, N.J.) mouse Igloo and Fast-Trac spinning enrichment devices and Nestlets pads (Ancare, Bellmore, NY). All animal procedures followed the National Research Council's “GUIDE FOR THE CARE AND USE OF LABORATORY ANIMALS” and were first reviewed and approved by the LSU IACUC.

F344 rats were purchased from Charles River Laboratories and arrived at 7 weeks of age. After a two-week period of quarantine, rats were identified by permanent marker on the tail. The rats were individually housed. Cage boxes were changed with fresh litter and fresh water weekly, and boxes were stocked with clean/autoclaved Crawl Balls enrichment devices (Bio-Serv, Frenchtown, N.J.).

### 2.4. Toxicity at 3- and 14-Day Time Periods in Mice

DCWL aloe juice was administered by gavage with a 20-gauge curved stainless steel gavage needle (Popper & Sons, New Hyde Park, NY) to male and female B6C3F1 mice twice over a 24-hour period at 1.0% of body weight. Seven mice per test group were gavaged either concentrated juice or water then observed daily for abnormal behaviors including hypoactivity, isolation from littermates, ataxia, dyspnea, or prostration. In addition, mice were monitored for a weight loss of 10% or greater, decreased feed consumption of 10% or greater, and death.

Through the oral gavage, female mice consumed an estimated amount of aloe vera juice equal to 32.1 times the amount a person would normally drink per 24-hour period. Male mice consumed an average of 27.8 times this amount. These estimates were determined by assuming the recommended daily amount of aloe juice a person drinks to be 8.0 ounces (236.6 mL). To scale this quantity to a mouse, we used body surface area ratio [27] comparing mice groups with a 5 feet, 10 inches person weighing 70 kg.

At the conclusion of the 3- and 14-day periods, gross necropsies were performed on all animals, and final body weights plus weights of the kidney (right), heart, lung, liver, and testes (males) were recorded. Hematologic parameters were assessed on blood samples collected in potassium EDTA containers. This analysis included RBC, Hb, HCT, RDW, MCV, MCH, MCHC, platelets, MPV, and total WBCs. Liver sections were screened independently by two pathologists. Finding of pathologic anomalies would trigger further microscopic evaluation of all organs collected from mice. Body weight, organ weights and all hematologic and clinical chemistry parameters were analyzed within each sex by Student's *t*-test, with significance as *P* ≤ 0.05.

### 2.5. Subchronic Toxicity in F344 Rats

Rats were divided into groups of 6 or 7. Control groups received AIM-93G chow (Teklab, Madison, WI) ad libitum while aloe-treated rats consumed chow formulated with concentrated aloe juice in place of water (Teklab, Madison, WI) for 13 weeks. Each week, weights of individual rats and feed consumed were recorded. Animals were assessed daily for general behavior, health, and appearance as well as appearance of stools.

At the conclusion of the 13-week study period, rats were anesthetized with isoflurane for blood collection and then humanely euthanized. At necropsy, general body appearance and external orifices were examined prior to examination of internal organs. Abdominal, pelvic, and thoracic organs were observed *in situ*, then the liver, kidneys, testes (in males), heart, and lungs were removed, weighed, rinsed with phosphate-buffered saline (PBS), and placed into 10% neutral buffered formalin (NBF). The intestinal tract was examined from the stomach distally, and then the tract from the cecum distally was labeled using colored thread for cecum, ascending, transverse, and descending colon and rectum. The large intestines were then removed, placed in PBS, and sectioned according to anatomic region. Intestinal contents were gently flushed with PBS, and using a blunt needle, the cecum was opened along the greater curvature and flushed and examined for gross lesions. The cecal-colic junction was opened and grossly examined. Each large intestinal region was preserved in 10% NBF. The small intestine was opened and grossly examined and preserved if lesions were observed. Fixed tissues were embedded in paraffin and sectioned at 7 um onto glass slides and stained with hematoxylin and eosin. Histological preparations and microscopic evaluations were made of the liver, cecum, and colon tissues (ascending, transverse, descending) and rectum. Each large intestine anatomic region was evaluated and scored using a semiquantitative scale in increments of 0.1 in which 0–0.9 indicated hypoplastic mucosa, 1.0–1.9 indicated mucosal architecture and cellular populations within expected ranges, 2.0–2.9 indicated moderate hyperplasia of the mucosal epithelia and increased lymphocytic infiltrate and/or goblet cell hyperplasia and secretion, and 3.0–3.9 indicated pronounced hyperplasia of mucosa and goblet cells, lymphoid aggregates with enlarged germinal centers, and inflammatory infiltrate. Mucosa of rats was independently scored by three reviewers, and the average scores for each large intestinal region were computed for aloe treatment and controls of each sex.

Feed consumption and body weight changes during the 13-week period were compared using two-way repeated measures ANOVA with Bonferroni post-tests. Organ weights were compared using an unpaired *t*-test with significance set a *P* ≤ 0.05. Hematologic tests included RBC, Hgb, HCT, RDW, MCV, MCH, MCHC, platelets, MPV, plasma protein, PCV, WBCs, neutrophils, lymphocytes, monocytes, and eosinophils. Clinical chemistry assays included glucose, AST, ALT, ALP, CK, T protein, albumin, globulin, cholesterol, BUN, creatinine, calcium, phosphorus, sodium, potassium, chloride, bicarbonate, and anion gap. Samples were compared with gender-matched controls using an unpaired *t*-test (*P* ≤ 0.05). Large intestinal mucosal thickness scores were compared using Mann-Whitney *U* ranking to obtain a *P* value set as *P* ≤ 0.05.

### 2.6. Verification of Aloe Juice Level in Feed

To verify DCWL aloe juice levels in the compounded feed, one juice constituent, malic acid, was determined in samples of 9.3x concentrated juice prior to compounding and in samples of the final aloe-containing chow. A Malate Assay kit (Biovision, Milpitas, California) was used to measure L(-) malate. The 9.3x aloe juice contained a measured 362.0 ± 18.0 mM malate; therefore, the chow was expected to contain a malate of 36.2 mM. The final measured malate concentration extracted from the finished chow was 37.8 ± 1.29 mM, or 104.4 ± 3.4% of the expected.

Male rats in this study consumed an average of 14.3 g aloe chow/day or 540.5 *μ*mol malate/day. Female rats consumed an average of 9.13 g of aloe chow/day or 344.0 *μ*mol malate/day. These malate levels indicate that the male rats exceeded a recommended 8.0 ounce human consumption by 5.9-fold and the female rats exceeded the human consumption of aloe juice by 5.1-fold based on body surface area.

## 3. Results

### 3.1. Genotoxicity

DCWL aloe vera juice extract when tested at up to a 21x concentration was not significantly mutagenic to test *Salmonella* spp. bacteria in the absence or presence of S9 liver extract. The blank plate without S9 showed a similar number of revertant wells in aloe plates of 21x, 14x, or 7x (Table 1(a)). Similarly, the blank plate with S9 did not show any significant differences from revertant wells in aloe +S9 plates of 21x, 14x, or 7x (Table 1(b)). The *P* value was ≥0.05 for all aloe juice concentrations, without and with S9. These statistics applied to both the TA100 and TA98 strains. This mutagenicity assay was developed sufficiently to be interpreted between days 3 and 5. A bacteria-free plate remained without growth indicating that there was no external bacterial contamination in the assay and positive controls for both sodium azide and 2-aminoanthracene plus S9 extract verified assay function (data not shown).

**Figure 1:**
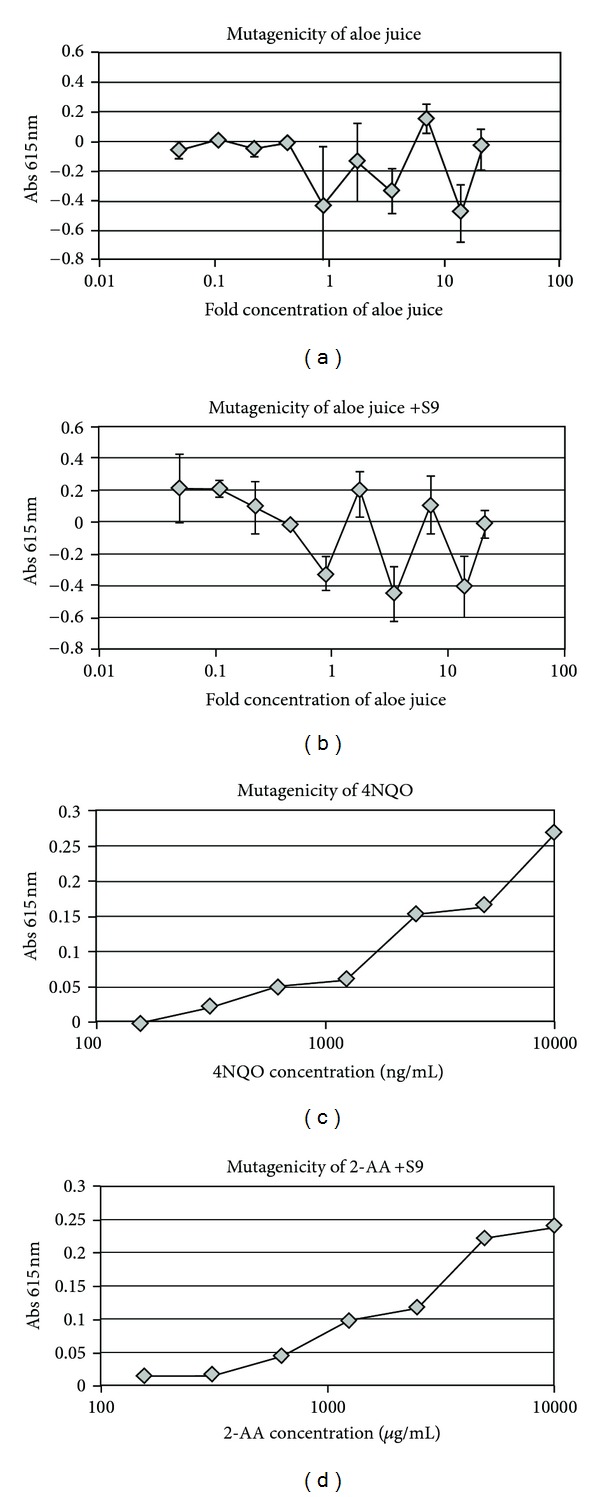
Effects of DCWL aloe vera juice on DNA damage repair. Absorbance reflects measurement of SOS transgene (*β*-galactosidase) induction and subsequent substrate conversion. Symbols represent means of triplicate wells incubated in the absence or presence of S9 rat liver extract. Error bars represent standard deviations.

SOS DNA repair in *E*. *coli* was not activated by DCWL aloe vera juice extract in concentrations ranging from 21-fold concentrated to approximately 20-fold diluted (Figure 1(a)). The juice product also did not show significant concentration-related DNA damage/repair in the presence of metabolically active liver extract (Figure 1(b)). These data indicate that juice does not contain significant levels of active or latent genotoxic compounds. SOS DNA repair was activated in the positive control 4-nitroquinoline oxide (4NQO) resulting in a concentration-dependent increase in DNA repair gene activity (Figure 1(c)). Bacteria also showed a concentration-dependent increase in DNA damage repair gene activity when exposed to the positive control chemical 2-aminoanthracene (2-AA) in the presence of metabolic extract from rat liver (Figure 1(d)).

### 3.2. Effects of Acute DCWL Aloe Administration in Mice at 3 and 14 Days Postadministration

There were no mortalities in any mice fed high concentrations of aloe juice. Mice were equivalent to control mice in behavior. Male and female mice after both 3 and 14 days postadministration of aloe had body and organ weights similar to control mice (Table 2). Necropsy was unremarkable in all mice.

Hematologic values from blood sampled from mice at 3 days and 14 days postexposure to the aloe vera juice were generally similar to control group mice (Table 3). The few values that appeared to be different between groups had very small variances within groups; thus, small differences between means were statistically calculated as different. However, in all cases the differences between means were 10% or less. Clinical chemistry values from blood sampled at these time periods were also similar to values for control group mice (Table 4).

Sections of liver from aloe-gavaged and control mice were microscopically examined. The architecture of the tissues and cellular morphology were considered typical of mice at their age (data not shown). For instance, male livers from both control and mice treated with the test extract showed moderate cytoplasmic vacuolation, instances of binucleate hepatocytes, and extramedullary hematopoiesis (EMH). The female mice treated with the test extract as well as controls showed multifocal EMH, some cytoplasmic vacuolation, and binucleate hepatocytes, but did not display the occasional mild hepatocellular necrosis seen in males. No pathologic features were determined to be exclusively or predominantly associated with test groups.

### 3.3. Subchronic Feed Administration, 13-Week Rat Studies

**Figure 2:**
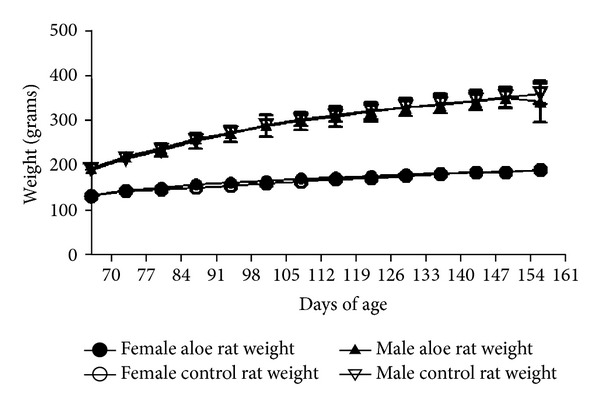
Weekly body weights of male and female rats fed DCWL aloe vera diet over 13 weeks. The symbols represent mean values for control and aloe-fed groups error bars show standard deviations.

**Figure 3:**
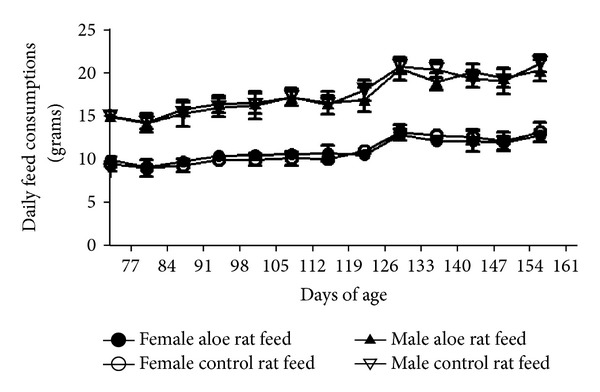
Weekly feed consumption of male and female rats fed DCWL aloe vera diet over 13 weeks. The symbols represent mean values for control and aloe-fed groups error bars show standard deviations.

During the course of study, there were no mortalities in any group. DCWL aloe vera-fed rats were equivalent to control rats in behavior. Both body weight gains and feed consumption of rats fed the test extract were equivalent to the same parameters in the control males and females (Figures 2 and 3).

One female rat died during transport from the vendor, and thus the control group of females contained 6 rats, while all other groups contained 7. One control female rat developed a facial swelling during the study. This swelling was diagnosed to be an abscessed tooth. The rat was treated with an antibiotic (enrofloxacin) and nonsteroidal anti-inflammatory (meloxicam) for a one-week period. This female responded well to therapy and was included in final data analyses.

At the study's conclusion, weights of organs were not significantly different from control in either sex (Table 5), and gross necropsy was otherwise unremarkable. Hematologic analyses showed that no parameter assessed in rats fed with the DCWL aloe vera differed significantly from the respective control group (Table 6). Clinical chemistry values from blood were also generally not different from control group mice (Table 7). There were no differences in males; however, in females, values for albumin and cholesterol were lower in rats fed the test extract versus control rats. Cholesterol was reduced in males fed the test extract, but this difference was not statistically significant.

The cecum (including the cecal-colic junction), ascending, transverse, and descending colon and rectum were opened and examined for gross abnormalities (i.e., masses or ulcerative lesions). No abnormalities were found in aloe or control rats of either gender.

## 4. Discussion

In this study, we sought to determine if oral administration of concentrated levels of a commercially available DCWL aloe juice, Lily of the Desert Filtered Whole Leaf Aloe vera Juice with Aloesorb, produced genotoxicity *in vitro*, acute/subacute toxicity in mice or subchronic toxicity in rats. In agreement with the results of others who have tested aloe vera from the inner gel fillet [18], we found no evidence to support genotoxic effects in bacterial assays.

In previously reported *in vivo* toxicity tests, aloe derivatives without the anthraquinone-containing latex are not associated with adverse effects. Ikeno et al. [28] tested dried powder from the inner leaf fillet in a feed study with Fischer 344 rats and found no toxicity. More recently, Tanaka et al. [29] reported no mortalities, no abnormalities at necropsy, and no differences in body weight gain after 14 days in a rat study. This study tested an aloe vera gel extracted with supercritical carbon dioxide administered as a single oral dose of 150 mg/kg. In our *in vivo* assays, we also found no evidence of aloe toxicity. In mice, our acute studies demonstrated no mortalities, no changes in behavior, body weight, or organ weights. Hematology and clinical chemistry values were generally not significantly different compared to controls although in some instances, a measured value for one parameter was different from the control at *P* ≤ 0.05. If the mouse showed no other subjective/objective change suggesting toxicity, we considered these values to be the occasional outliers, which would be anticipated given the large number of individual parameters quantitated, and we did not feel that these were biologically relevant differences between aloe-treated and untreated groups.

Rats fed DCWL aloe vera juice over 13 weeks also displayed no adverse signs. Necropsy evaluations, hematology, and clinical chemistries were generally similar to the control groups. The difference in plasma albumin was not felt to be a toxic effect since we found no differences in total protein, globulin, or serum protein in females and because albumin in males fed with the test extract was not different (*P* = 0.94). One notable effect was the significantly reduced cholesterol concentration in females. Cholesterol has been reported to be decreased in rats that were administered aloe vera previously [30, 31]; thus, this result in our analysis is not surprising. Huseini et al. [7] have reported that aloe gel reduced cholesterol in humans in a clinical trial; however, these clinical results have not been verified in other studies to date. This was a relatively short-termed study with young, healthy animals, and this effect may have been more pronounced and/or seen in males over a longer term or in aged animals. Studies employing innately hypercholesterol rat models have shown a greater cholesterol-reducing effect of aloe [32]. Natural products that lower cholesterol are sought after by consumers of supplements. Therefore, although this study was not one of efficacy determination, it is a notable observation.

Of particular interest, in our 13-week rat study, there was an analysis of the large intestine for signs of mucosal pathology. These pathologies were reported in F344 rats with water-administered, nondecolorized whole-leaf aloe vera extract [10] and featured a significantly increased incidence and severity of mucosal hyperplasia with goblet cell hyperplasia that was more pronounced in male rats. Our data obtained using a DCWL aloe vera juice contrast with these previous results. No gross or microscopic evidence of intestinal pathologies was observed. Rat intestinal sections from both control and rats fed with the test extract displayed typical mucosal phenotypes including sections with variable levels of focal lymphoid cellularity in the lamina propria and occasional variations in the degree of fibrous tissue between crypts; however, crypts were uniform in length and depth and goblet cells were evenly distributed and not over productive with mucous secretions.

The differences in findings between our present study and that of the NTP are most likely due to the different aloe vera juices tested. We have not identified specific components in nondecolorized whole-leaf aloe extract that are reduced or absent in purified whole leaf beverages and which may have led to the mucosal response observed by the NTP. However, attractive candidates are the anthraquinones associated with the latex in whole leaf juice but largely absent from activated charcoal-treated aloe. Most aloe anthraquinones exist as glycosides, which are rarely mutagenic [33]. Free anthraquinones, released in animals after intestinal microbial oxidation of the glycosidic bonds, have been associated with *in vitro* mutagenesis, although aloe-emodin, the predominant anthraquinone released by microbial action on aloin glycosides, has been repeatedly found to be negative in a variety of *in vivo* assays (reviewed by [31]). Longer termed animal studies, particularly with a sensitive species, could reveal toxicologic or carcinogenic effects not seen with acute assays, and the rat model seems well suited to potentiate toxic and carcinogenic effects of aloe's anthraquinones. Rats (as well as mice) have substantial quantities of microbial flora throughout their upper as well as lower gastrointestinal tracts [34] including those such as *Eubacterium* spp. which are capable of oxidizing the barbaloin C-glycosidic bond [35, 36]. In contrast, humans have a relatively sterile upper GI tract [32], and anthroid glycosides pass intact to the colon before limited C-glycosidase activity occurs [31]. Other aloe vera components, such as polysaccharides that serve as growth substrates for *Eubacterium* [37], could alter intestinal flora in favor of glycosidase-capable bacteria. The rat may therefore be an ideal sentinel for intestinal toxicities associated with the phenolics in aloe; however, extrapolation of carcinogenic outcomes should account for the degree of relative human exposure to the free anthraquinones and the levels of anthraquinone glycosides in the aloe juice extract tested.

The levels of aloe vera consumed by rats in the present study are similar to a concentration consumed by rats in the 2-year rat NTP study (see Table 8). The NTP study used dried nondecolorized whole leaf aloe juice powder at levels of 0, 0.5, 1.0, and 1.5% in drinking water. At the 1.0% level, male rats in the NTP study drank an average of 28.80 mls/day at approximately the 4-week time point. The drinking water contained a target of 1980 ppm of malic acid. This equates to 212.8 *μ*mol malate/day/100 g rat body weight. Females consumed a similar amount. In our study, males and females consumed approximately 232 *μ*mol malic acid/day/100 g weight. These data indicate that the rats in our study consumed an amount of aloe juice equivalent to the 1.0% aloe drinking water in the NTP study by malic acid equivalency.

In summary, after assessing a DCWL aloe beverage for both genetic and *in vivo* toxicity, we found no adverse effects associated with high intake of aloe at acute or subchronic periods. Importantly, we did not find hyperplastic mucosal changes that were so notable in a recent report using nondecolorized whole-leaf aloe. Therefore, it is reasonable to believe that components in the untreated leaf extract are responsible for the large intestinal hyperplastic reactions at 13 weeks.

## Figures and Tables

**Table:** (a)

Sample	No. of positive wells @ 2 days	No. of positive wells @ 3 days	No. of positive wells @ 4 days	No. of positive wells @ 5 days
Blank without S9	0	3	3	5
Blank + S9	0	3	11	16
WL Aloe 21x	0	0	0	0
WL Aloe 21x + S9	0	2	0	0
WL Aloe 14x	0	0	0	0
WL Aloe 14x + S9	0	0	0	10
WL Aloe 7x	0	2	4	9
WL Aloe 7x + S9	0	0	1	6
WL Aloe 7x + 2x S9	0	2	3	8

@ indicates at.

**Table:** (b)

Sample	No. of positive wells @ 2 days	No. of positive wells @ 3 days	No. of positive wells @ 4 days	No. of positive wells @ 5 days
Blank without S9	0	0	1	2
Blank + S9	0	3	17	20
WL Aloe 21x	0	0	0	3
WL Aloe 21x + S9	0	0	15	17
WL Aloe 14x	0	0	2	4
WL Aloe 14x + S9	0	0	14	16
WL Aloe 7x	0	0	0	0
WL Aloe 7x + S9	0	0	3	11
WL Aloe 7x + 2x S9	0	0	10	15

**Table 2:** Selected organ weights of B6C3F1 mice administered aloe juice by gavage.

3 days post aloe	Male control	Aloe male	Control female	Aloe female
Mortalities	0/6	0/6	0/6	0/6
Abnormal clinical signs	0/6	0/6	0/6	0/6
Gross abnormalities	0/6	0/6	0/6	0/6
Weight change % init. b.wt.	0.115 ± 1.03	0.868 ± 2.15	4.12 ± 2.83	3.87 ± 2.05
Relative organ weight^*∧*^				
Liver relative weight	4.58 ± 0.240	4.35 ± 0.113	5.01 ± 0.173	4.97 ± 0.315
Kidney (rt)	0.769 ± 0.072	0.750 ± 0.029	0.724 ± 0.047	0.679 ± 0.048
Heart	0.460 ± 0.046	0.473 ± 0.044	0.480 ± 0.025	0.498 ± 0.056
Lung	0.592 ± 0.088	0.578 ± 0.062	0.667 ± 0.033	0.663 ± 0.069
Testicle (rt)	0.390 ± 0.044	0.371 ± 0.017	—	—

14 days post aloe				

Mortalities	0/6	0/6	0/6	0/6
Abnormal clinical signs	0/6	0/6	0/6	0/6
Gross abnormalities	0/6	0/6	0/6	0/6
Weight Change % init. b.wt.	3.36 ± 2.91	6.37 ± 0.772	7.18 ± 2.65	6.85 ± 3.46
Relative organ weight^*∧*^				
Liver	4.85 ± 0.252	4.65 ± 0.108	5.21 ± 0.372	4.74 ± 0.388
Kidney (rt)	0.769 ± 0.072	0.750 ± 0.029	0.724 ± 0.047	0.679 ± 0.048
Heart	0.487 ± 0.077	0.442 ± 0.037	0.585 ± 0.063	0.482 ± 0.061
Lung	0.633 ± 0.080	0.614 ± 0.650	0.725 ± 0.075	0.662 ± 0.053
Testicle (rt)	0.390 ± 0.044	0.371 ± 0.017	—	—

Values presented as mean ± s.d.

^*∧*^Relative organ weights are expressed as a percent of body weight.

**Table 3:** Hematology of whole blood from B6C3F1 mice administered aloe juice by gavage.

Parameters—3 days post exposure	Control male	Aloe male	Control female	Aloe female
RBC (10^6^/uL)	8.6 ± 0.21	8.8 ± 0.18	8.7 ± 0.26	8.8 ± 0.25
Hb (g/dL)	13.1 ± 0.33	13.4 ± 0.28	13.3 ± 0.33	13.4 ± 0.31
HCT (%)	43.5 ± 1.29	44.1 ± 1.16	43.2 ± 1.34	42.9 ± 1.21
RDW (%)	12.7 ± 0.45	12.7 ± 0.30	12.3 ± 0.46	11.9 ± 0.36
MCV (fL)	50.4 ± 0.48	50.2 ± 0.86	49.6 ± 0.30	48.7 ± 0.44*
MCH (fL)	15.2 ± 0.04	15.3 ± 0.18	15.4 ± 0.18	15.2 ± 0.16
MCHC (g/dL)	30.1 ± 0.23	30.4 ± 0.32	31.0 ± 0.43	31.2 ± 0.27*
Platelets (10^3^/uL)	1100.0 ± 102.10	1116.8 ± 129.31	952.2 ± 128.94	1041.7 ± 128.75
WBC's (10^3^/uL)	2.2 ± 1.02	3.3 ± 2.86	2.1 ± 0.92	2.3 ± 0.58

Parameters—14 days post exposure				

RBC (10^6^/uL)	8.7 ± 0.32	8.7 ± 0.27	8.4 ± 0.28	8.7 ± 0.35
Hb (g/dL)	13.2 ± 0.39	13.1 ± 0.41	13.1 ± 0.64	13.6 ± 0.42
HCT (%)	44.7 ± 2.03	43.8 ± 1.21	42.7 ± 0.87	43.3 ± 1.48
RDW (%)	13.0 ± 0.43	13.2 ± 0.64	14.0 ± 3.53	12.2 ± 0.71
MCV (fL)	51.0 ± 0.75	50.2 ± 0.45*	51.7 ± 3.54	49.6 ± 0.28
MCH (fL)	15.1 ± 0.18	15.0 ± 0.08	15.8 ± 0.19	15.6 ± 0.14
MCHC (g/dL)	29.5 ± 0.50	29.8 ± 0.36	30.6 ± 1.6	31.5 ± 0.14
Platelets (10^3^/uL)	1068.8 ± 129.2	1100.0 ± 104.5	955.2 ± 310.53	1023.5 ± 16.2
WBC's (10^3^/uL)	4.2 ± 1.5	3.8 ± 1.1	3.1 ± 2.3	2.4 ± 0.42

Values expressed as mean ± s.d.

*P ≤ 0.05.

RBC: red blood cell count; Hb: hemoglobin; HCT: hematocrit; MCV: mean corpuscular volume; MCH: mean corpuscular hemoglobin; WBC: total white blood cell count; RDW: red blood cell distribution width; MCHC: mean corpuscular hemoglobin concentration; WBC's: white blood cell count.

**Table 4:** Clinical chemistry of plasma from B6C3F1 mice administered aloe juice by gavage.

Parameters—3 days post exposure	Control male	Aloe male	Control female	Aloe female
ALT (U/L)	26.0 ± 3.5	23.0 ± 5.4	18.4 ± 2.0	19.7 ± 2.2
ALP (U/L)	64.2 ± 4.4	60.56 ± 7.3	87.8 ± 9.4	88.8 ± 16.4
CK (U/L)	287.6 ± 264.8	146.0 ± 114.7	85.4 ± 29.2	120.2 ± 68.3
TBIL (mg/dL)	≤0.1^#^	≤0.1	≤0.1	≤0.1
Total protein	4.5 ± 0.23	4.3 ± 0.1*	4.1 ± 0.13	4.0 ± 0.42
BUN (mg/dL)	18.0 ± 1.2	18.0 ± 1.4	22.8 ± 1.9	22.5 ± 1.1
Creatinine (mg/dL)	≤0.2^#^	≤0.2	≤0.2	≤0.2

Parameters—14 days post exposure				

ALT (U/L)	25.2 ± 7.4	21.8 ± 6.5	23.2 ± 11.9	18.7 ± 2.4
ALP (U/L)	71.2 ± 18.0	67.4 ± 5.0	101.3 ± 3.9	86.2 ± 16.6
CK (U/L)	117.0 ± 14.3	169.2 ± 86.9	157.2 ± 105.5	196.0 ± 144.6
TBIL (mg/dL)	≤0.1^#^	≤0.1	≤0.1	≤0.1
Total protein	3.8 ± 0.06	4.0 ± 0.3	3.8 ± 0.03	3.8 ± 0.2
BUN (mg/dL)	20.8 ± 4.0	21.8 ± 2.6	24.3 ± 2.3	22.2 ± 1.0
Creatinine (mg/dL)	≤0.2^#^	≤0.2	≤0.2	≤0.2

Values expressed as mean ± s.d.

*P ≤ 0.05.

ALT: alanine aminotransferase activity; ALP: alkaline phosphatase Activity; CK: creatine kinase; TBIL: total bilirubin; BUN: blood urea nitrogen.

^#^Levels for TBIL and creatinine were at or below instrument quantitation threshold.

**Table 5:** The effect of aloe vera administration for 13 weeks on rat organ weights.

Parameter	Male control	WL aloe male	Control female	WL aloe female 13 week
Mortalities	0/7	0/7	0/6	0/7
Abnormal clinical signs	0/7	0/7	1/6	0/7
Gross abnormalities at necropsy	0/7	0/7	0/6	0/7
% body weight change	190.3 ± 6.63	177.5 ± 23.10	143.6 ± 5.82	144.4 ± 10.74
Relative organ weight^*∧*^				
Liver	3.76 ± 0.233	3.82 ± 0.178	3.30 ± 0.103	3.28 ± 0.164
Kidney (rt)	0.338 ± 0.059	0.374 ± 0.070	0.334 ± 0.045	0.328 ± 0.072
Kidney (lf)	0.334 ± 0.077	0.412 ± 0.098	0.352 ± 0.049	0.336 ± 0.061
Heart	0.325 ± 0.034	0.294 ± 0.050	0.419 ± 0.051	0.376 ± 0.044
Lung	0.490 ± 0.081	0.550 ± 0.080	0.540 ± 0.059	0.497 ± 0.082

All values expressed as mean ± standard deviation.

*P ≤ 0.05 compared with gender control.

^*∧*^Relative organ weights are expressed as a percent of body weight.

**Table 6:** The effect of aloe vera administration for 13 weeks on rat hematologies.

Parameter	Control male	WL aloe male	Control female	WL aloe female
RBC (10^6^/uL)	7.95 ± 0.28	8.28 ± 0.34	7.26 ± 0.59	6.94 ± 0.42
Hgb (g/dL)	12.54 ± 0.32	12.94 ± 0.50	12.32 ± 0.20	11.80 ± 0.71
HCT (%)	38.76 ± 1.13	40.09 ± 1.33	37.68 ± 1.21	35.83 ± 1.97
RDW (%)	12.99 ± 0.30	12.89 ± 0.11	8.10 ± 5.08	11.29 ± 0.33
MCV (fL)	48.73 ± 0.54	48.66 ± 0.59	51.05 ± 0.67	51.60 ± 0.97
MCH (fL)	15.79 ± 0.22	15.73 ± 0.14	16.67 ± 0.45	17.00 ± 0.77
MCHC (g/dL)	32.40 ± 0.23	32.99 ± 0.37	32.67 ± 0.58	32.94 ± 0.52
Platelets (10^3^/uL)	613.43 ± 48.41	586.57 ± 66.12	569.17 ± 84.41	564.43 ± 61.43
MPV (fL)	7.67 ± 0.61	7.40 ± 0.13	7.72 ± 0.30	7.99 ± 0.51
Plasma protein (g/dL)	7.56 ± 0.61	12.03 ± 13.22	6.80 ± 0.46	6.47 ± 0.69
PCV (%)	38.43 ± 1.13	39.29 ± 1.60	36.17 ± 0.98	34.71 ± 1.60
WBC's (10^3^/uL)	4.73 ± 0.56	5.17 ± 1.19	3.42 ± 0.53	3.07 ± 1.09
Neutrophils (%)	0.89 ± 0.27	0.93 ± 0.24	0.52 ± 0.10	0.61 ± 0.23
Lymphocytes (%)	3.59 ± 0.69	3.96 ± 0.95	2.67 ± 0.49	2.31 ± 0.90
Monocytes (%)	0.14 ± 0.05	0.21 ± 0.11	0.15 ± 0.05	0.13 ± 0.10
Eosinophils (%)	0.07 ± 0.05	0.06 ± 0.05	0.03 ± 0.05	0.04 ± 0.05

All values expressed as mean ± standard deviation.

*P ≤ 0.05 compared with gender control.

**Table 7:** The effect of aloe vera administration for 13 weeks on rat clinical chemistires.

Parameter	Control male	WL aloe male	Control female	WL aloe female
Glucose (mg/dL)	221.0 ± 36.13	200.7 ± 59.92	190.3 ± 8.26	168.9 ± 34.28
AST (U/L)	61.14 ± 14.50	62.33 ± 8.45	71.17 ± 18.98	68.14 ± 12.27
ALT (U/L)	35.29 ± 5.22	41.67 ± 13.95	29.33 ± 10.29	25.86 ± 4.45
ALP (U/L)	147.0 ± 15.77	152.8 ± 5.91	167.17 ± 12.58	150.43 ± 28.81
CK (U/L)	190.43 ± 113.15	353.00 ± 380.35	204.50 ± 72.02	235.29 ± 132.17
Total protein (g/dL)	5.81 ± 0.47	6.02 ± 0.43	5.67 ± 0.45	5.25 ± 0.52
Albumin (g/dL)	3.06 ± 0.24	3.07 ± 0.16	3.23 ± 0.27	2.88 ± 0.23*
Globulin (g/dL)	2.76 ± 0.26	2.95 ± 0.28	2.42 ± 0.23	2.37 ± 0.31
Cholesterol (mg/dL)	103.7 ± 17.21	94.83 ± 6.85	102.8 ± 9.95	84.00 ± 12.81*
BUN (mg/dL)	21.29 ± 2.06	20.17 ± 1.60	17.33 ± 7.94	18.86 ±1.46
Creatinine (mg/dL)	0.31 ± 0.035	0.32 ± 0.032	0.34 ± 0.02	0.30 ± 0.03
Calcium (mg /dL)	8.99 ± 0.69	9.37 ± 0.67	8.25 ± 1.26	7.46 ± 0.94
Phosphorus (mg/dL)	3.89 ± 0.41	4.00 ± 0.44	3.57 ± 0.38	3.03 ± 0.55
Sodium (mmol/L)	141.1 ± 1.22	140.7 ± 1.51	140.6 ± 1.97	142.1 ± 1.57
Potassium (mmol/L)	3.24 ± 0.30	3.50 ± 0.18	2.75 ± 0.14	2.59 ± 0.20
Chloride (mmol/L)	103.3 ± 3.15	101.8 ± 2.32	106.2 ± 2.14	110.6 ± 4.61
Bicarbonate (mmol/L)	25.00 ± 2.00	26.57 ± 2.70	23.2 ± 3.63	20.49 ± 2.68
Anion gap (mmol/L)	16.11 ± 1.50	15.77 ± 0.77	13.73 ± 1.67	13.67 ± 1.51

All values expressed as mean ± standard deviation. *P ≤ 0.05 compared with gender control.

**Table 8:** Consumption of Aloe vera by rats in this study versus NTP drinking water study.

Doses of aloe	Target malic acid concentration	Malic acid molarity in dosed form (mM)	Consumption of dosed form (g/day)*	Weight of rats at 4 weeks (g)*	umol/day malic acid consumed/100 g body weight
NTP 1% in water	1980 (ppm)^*∓*^	14.8	28.80 male 22.46 female	200.0 male 142.6 female	212.8 male 232.8 female
Present study 100 g/kg	36.2 mmol/kg feed^*∧*^	37.8^#^	14.30 male 9.13 female	239.0 male 150.0 female	235.0 male 229.3 female

^∓^Taken from Table H4, Target values for malic acid administered to rats in the 2-year study at approximately 4 weeks, pg. 260 NTP study.

^*∧*^Measured in Aloe feed extract.

*NTP data obtained from Tables J1 and J2, pgs. 283 and 284 NTP study.

^#^Assumes 1 gram feed = 1 ml solid.

## Abbreviations

RBC: Red blood cell count
Hb: Hemoglobin
HCT: Hematocrit
MCV: Mean corpuscular volume
MCH: Mean corpuscular hemoglobin
WBC: Total white blood cell count
RDW: Red blood cell distribution width
MCHC: Mean corpuscular hemoglobin concentration
PCV: Packed cell volume
ALT: Alanine aminotransferase activity
ALP: Alkaline phosphatase activity
CK: Creatine kinase
TBIL: Total bilirubin
BUN: Blood urea nitrogen
PPM: Parts per million
DCWL: Decolorized whole leaf
2-AA: 2-aminoanthracene
4NQO: 4-Nitroquinoline 1-oxide.
